# Phytomelatonin as a key regulator of drought stress adaptation: signaling networks, microbial interactions, and biotechnological potential

**DOI:** 10.3389/fpls.2026.1817297

**Published:** 2026-05-21

**Authors:** Namo Dubey, Shubhangi Shree, Sneha Prakash, Vaishnavi Subramanian, Syama H.P., Subhash Kumar

**Affiliations:** 1 School of Bio Sciences and Technology, Vellore Institute of Technology, Vellore, Tamil Nadu, India; 2 Department of Integrative Biology, School of Bio Sciences and Technology, Vellore Institute of Technology, Vellore, Tamil Nadu, India; 3 Department of Bio Medical Sciences, School of Bio Sciences and Technology, Vellore Institute of Technology, Vellore, Tamil Nadu, India

**Keywords:** drought, metabolic engineering, phytohormone, phytomelatonin, plant growth

## Abstract

The impact of drought stress has remained a major problem that affects crop yield, growth, and development worldwide. There is increasing research interest showing that phytomelatonin acts as an integrative regulator that enhances plant tolerance to drought. Phytomelatonin, which is a potent antioxidant, acts by scavenging the reactive oxygen species (ROS) in plants but its role in drought tolerance is greater than antioxidation. It is of key importance for several physiological and developmental processes involved in plant survival, including seed germination, root development, and leaf senescence. The various pathways in which phytomelatonin mediate drought tolerance are multifaceted and involve molecular mechanisms of signaling. These pathways involve interactions with membrane-bound receptor proteins, notably phytomelatonin receptor 1 (PMTR1), the activation of downstream signaling components like mitogen-activated protein kinase (MAPK) cascades, and various transcription factors that regulate gene expression in response to stress. In this review the molecular mechanisms of phytomelatonin treatment under drought stress have been explored. In addition, it explores innovative approaches to enhance the precision of genetic and metabolic engineering strategies through the application of melatonin, aiming to improve drought tolerance of plants in a comprehensive manner. Melatonin-mediated resistance to drought, partly through activation of plant-associated microbial communities, shows promising effects. However, the underlying mechanisms of how melatonin influences these microbial communities under drought stress remain poorly understood. This review consolidates the research conducted on melatonin, emphasizing this developing field of study. Additionally, it underscores the significance of melatonin’s interactions with other phytohormones in facilitating adaptation to drought conditions, aiming to enhance its regulatory functions. Drawing from the aforementioned developments, this review provides a summary of recent advances to systematically explore the impact of melatonin on drought tolerance and describes research directions to improve the understanding of its various implications and applications in sustainable agricultural systems in drought-affected regions.

## Introduction

Drought is one of the most serious environmental challenges affecting agriculture worldwide. The driving factors are generally the change of precipitation patterns, rising temperatures, and inappropriate drainage systems (Wang and Ren, 2025). Under drought stress, plants accumulate abundant reactive oxygen species (ROS) that can damage cellular structures (Ahammed et al., 2024). However, there are also natural defense mechanisms in plants such as antioxidant systems that help to reduce ROS. Nevertheless, they may not continue in extreme or extended stress, leading to reduced photosynthetic activity and decreased plant growth (Cannea and Padiglia, 2025; Dubey et al., 2025). As a result, research has increased to identify phytoprotectants that can improve plant drought stress resilience. Conversely, the shortage of water reduces membrane integrity, thus adversely impacting photosynthetic performance and the potential crop yield (Muhammad et al., 2021). In the plant biology field, how plants perceive and respond to drought remains the core question since drought is detrimental to plant survival and development. Despite the relatively limited understanding in the role of phytomelatonin in plant stress responses, it has taken a prominent position during the last decades, emerging as an effective modulator for plant response towards abiotic stress (Wang L. et al., 2017; Khan et al., 2024). Extensive systematic studies have shown that exogenous melatonin treatment improves drought tolerance in plants since 2013, relying on the previous knowledge suggesting the protective role of melatonin under stress (Wang et al., 2013). Phytomelatonin, a plant hormone that exhibits structural similarities with its animal analogue, has recently gained attention in promoting plant resistance and drought performance (Arnao and Hernández-Ruiz, 2020; Chen et al., 2025). The most significant antioxidant activity is scavenging of the free radicals, ROS, reactive nitrogen species (RNS), and other oxidative agents. Phytomelatonin has antioxidant activity but also plays a role in facilitating plant growth and development and significantly increasing the tolerance against drought (Luo et al., 2024). Colombage et al. (2023) reported that melatonin treatment increased crop yield by approximately 20% under multiple stresses, including salinity, heat, and drought, and by about 18% under drought conditions alone compared to untreated plants. Furthermore, plants treated with melatonin showed a 44% higher photosynthetic efficiency compared to the untreated control plants under drought stress, which enhances plant yield performance (Yang et al., 2023). The balance between the effects of melatonin and drought stress has been described as a cascading influence on plant stress on diverse physiological, biochemical, and molecular mechanisms interacting to enhance plant stress resilience. Phytomelatonin is also important for maintaining the cellular water balance and reducing oxidative damage, as well as regulating the expression of genes that respond to stress (Sati et al., 2024). It is said to exert protective roles in hormone regulation and antioxidant defense pathways promoting drought stress adaptation in plants (Zhao and Hu, 2023). Therefore, phytomelatonin is no longer considered as a circadian regulator; it is now recognized as a versatile signaling molecule that mediates plant–environment interactions (Khan et al., 2024; Siddiqui et al., 2019). Phytomelatonin under drought stress has been investigated in the context of a rapidly growing area of research that goes far beyond traditional plant hormones. It shows the advanced molecular strategies that plants use to respond to variable and extreme environments. The objective of this review is to summarize existing research on phytomelatonin in plants, contribute to current knowledge, and provide insights into its potential role in improving plant drought tolerance under natural conditions. Phytomelatonin plays an important role in plant biotechnology through various physiological and molecular mechanisms. Melatonin’s role in plant development, its interaction with phytohormones, and its prospective uses in molecular engineering methods with respect to drought-resistance will be explored in the following sections.

## Melatonin-mediated modulation of plant growth and development in response to drought stress

Drought stress disrupts photosynthesis, cell expansion, and nutrient metabolism; however, melatonin accumulation or exogenous application of melatonin alleviates these adverse effects by maintaining the chlorophyll metabolism system, physiologic equilibrium, enhancing antioxidant capacity, maintaining cellular homeostasis, and modulating stress-responsive gene expression (Arnao and Hernández-Ruiz, 2015; Zhang et al., 2014). One of the main roles of phytomelatonin is the maintenance of chlorophyll content by inhibiting its degradation (Zeng et al., 2022). Under water scarcity conditions, melatonin contributes to the stabilization of PSII and supports effective electron transport by enhancing antioxidant defenses. These defenses protect thylakoid membranes from ROS–induced damage and minimize photoinhibition (Liang et al., 2019). In rice, melatonin application has been reported to improve root and shoot growth, chlorophyll stability, relative water content, and PSII efficiency during drought stress. These physiological improvements are accompanied by the upregulation of drought- and stress-responsive genes such as *DREB*, *HSF*, and *NHX*, as well as enhanced activities of antioxidant enzymes including superoxide dismutase, catalase, and ascorbate peroxidase (Li et al., 2017; Wei et al., 2015). Similarly, in maize (*Zea mays*), melatonin application has been found to influence root architectural plasticity through auxin-related signaling pathways, maintains stomatal conductance, and enhances osmolyte accumulation, thereby supporting sustained photosynthesis and biomass production under drought stress (Li et al., 2021). Upon treatment of melatonin on sugar beet seedlings, the growth phenotype, biomass and plant height changes showed optimal effect on the concentration of 100 µM·L^−1^ under drought stress (He et al., 2023). Similarly, exogenous application of melatonin to peach *(Prunus persica*) seedlings has significantly raised chlorophyll content and photosynthetic efficiency to improve growth and minimize oxidative damage under drought conditions (Naghizadeh et al., 2024). Melatonin treatment resulted in higher water potential, higher CO_2_ absorption, elevation of chlorophyll a and total phenolics levels in leaf in highbush blueberry (*Vaccinium corymbosum* L.) compared to untreated plants under drought (Sandoval et al., 2024). 100 μM melatonin application on fenugreek, stevia, surinam cherry, pea plants, tomato seedlings, and barley significantly increased transpiration rate, net photosynthesis, antioxidant enzyme activities, and decreased chlorophyll breakdown (Amiri et al., 2024; Sheikhalipour et al., 2023; Labulo et al., 2024; Dzinyela et al., 2024). Also, exogenous application of melatonin enhances drought tolerance in diverse plant species, resulting in improved growth performance and yield (**Table 1**). Besides, leaf relative water content (RWC) is a key factor that shows the survival capability and leaf water level of the plants. Melatonin treatment resulted in a significant increase in RWC in maize under drought stress. Moreover, melatonin modulates phytohormone signaling, particularly by interacting with abscisic acid metabolism and signaling pathways, thereby fine-tuning stomatal regulation and water-use efficiency during drought (Li et al., 2015; Arnao and Hernández-Ruiz, 2019).

**Table 1:** Role of exogenous melatonin in mitigation of drought stress in plants.

Plant species	Effective melatonin concentrations	Mechanistic insight	Reference
*Arabidopsis thaliana*	50 µM	• Induced the expression of stress-responsive genes, accompanied by an increased accumulation of soluble sugars	Moustafa-Farag et al., 2020
*Oryza sativa*	100 µM	Promoted plant growth, increased the accumulation of osmoprotectants such as proline, improved mitochondrial structural integrity, activated stress-responsive gene expression, and reduced reactive oxygen species (ROS) levels as well as electrolyte leakage	Moustafa-Farag et al., 2020
*Solanum lycoperscium*	100-200 µM	• Reduces lipid peroxidation and membrane damage • Improved PSII efficiency, improved chlorophyll and better activity of antioxidant enzymes	Liu et al., 2015; Karaca and Cekic, 2019; Ibrahim et al., 2020
*Zea mays*	~100 µM	• Strengthens antioxidant capacity • Maintains stomatal conductance • Improves photosynthetic performance • Reduces oxidative injury to cellular structures	Ye et al., 2016
*Triticum aestivum*	500 µM	• Lower membrane damage, support grana lamella, higher efficiency of PS II	Cui et al., 2018
*Hordeum vulgare*	1 mM	• Higher endogenous melatonin, enhanced ABA synthesis, better water status, photosynthesis, antioxidants, PSII efficiency	Moustafa-Farag et al., 2020
*Jinyu Chuju (Dendranthma Morifolium)*	~100 µM	• Improved chlorophyll, photosynthesis, improved biomass, cell membrane damage, osmoprotectants (TSS and proline), enhanced relative conductivity, reduced MDA	Moustafa-Farag et al., 2020
*Camellia sinensis*	100 µM	• Better photosynthetic efficiency, higher the antioxidant enzymes activity	Bose and Howlader, 2020
*Pennisetum glaucam*	100-150 µM	• Enhances water use efficiency under stress conditions • Boosts antioxidant enzyme activity • Increases proline accumulation • Manages reactive oxygen species levels • Reduces electrolyte leakage • Lowers lipid peroxidation levels	Awan et al., 2023
*Glycine Max*	100 µM	• Enhances stress resilience • Regulates cellular redox homeostasis • Increases osmolyte accumulation • Activates stress-responsive transcription factors • Sustains overall physiological performance	Alam et al., 2018

Melatonin stimulates cell division and elongation through regulation of cell cycle–related genes, facilitating continued vegetative growth in drought conditions (Shi et al., 2024). Melatonin also facilitates reproductive development, increasing pollen viability, pollen germination, and fertilization effectiveness. This leads to increase in grain numbers per panicle, as well as proper grain filling under drought conditions (Wang et al., 2022a). Melatonin regulates expression of transport and assimilation genes including *NRT1.1* and *AAP1*, and thus promotes effective allocation of nutrients to the reproductive organs and maintenance of yield and grain quality, enhancing crop resilience and yield stability in drought-prone environments (Sharif et al., 2018). Apart from this, the melatonin treatment improved grain quality by moderate gluten accumulation. Likewise, melatonin supplementation in tea (*Camellia sinensis* L.) seedlings was shown to considerably improve drought resistance as evidenced by the decreased membrane damage, increased proline, total proteins and sugars levels, and augmented antioxidant enzyme activity (such as catalase, peroxidase) across multiple cultivars under drought stress (Langaroudi et al., 2023). In soybean (*Glycine max*), melatonin treatment significantly reduces hydrogen peroxide and malondialdehyde accumulation while increasing antioxidant enzyme activities and glutathione content under drought stress. These responses contribute to improved membrane stability, higher photosynthetic efficiency, and enhanced biomass accumulation (Wei et al., 2015; Imran et al., 2021). Exogenous treatment of melatonin in foxtail millets has found to preserve the functionality of photosynthetic organs and maintain the stability of photosynthetic pigments through which it can mitigate the drought-induced suppression of photosynthetic performance (Ren et al., 2025).

By maintaining redox homeostasis, melatonin protects cellular components from oxidative damage and supports sustained cell division and elongation under drought stress (Reiter et al., 2015). Melatonin has been reported to promote root elongation, nitrogen metabolism, and osmotic balance among chickpea and other legumes showing a simultaneous response to regulating growth and stress adaptation (Gajardo et al., 2025). Previous studies indicated that melatonin may be involved in drought stress priming and stress memory in plants. Melatonin-primed plants also recover faster, have more extensive antioxidant activity, and show greater growth upon recurrent drought exposure, perhaps related to prolonged transcriptional activation of stress-responsive genes and epigenetic regulation (Park et al., 2025).

## Enhancement of osmolyte accumulation and maintenance of cellular homeostasis

Drought stress causes osmotic imbalance involving cellular dehydration, loss of turgor pressure, and alterations in metabolic processes (Arnao and Hernández-Ruiz, 2015; Reiter et al., 2015). Melatonin has been reported to modulate osmolytes accumulation in plants by regulating metabolic pathways involved in the synthesis of compounds such as glycine betaine, proline, and soluble sugars which are osmoprotectants to increase the retention of water and osmotic balance in drought conditions (Colombage et al., 2023). In the previous study, melatonin induces the priming effect on alfalfa (*Medicago sativa*), effectively inducing drought resistance by correcting the osmoprotective mechanisms and regulating nitro-oxidative homeostasis (Sun et al., 2021). Foliar melatonin supplementation in three *Capsicum* species (*C. chinense, C. frutescens* and *C. annuum*) in 0 and 50 μM concentrations produced positive, but species-specific, responses, increasing the production of ions and supporting plant growth. Prolonging the osmolytes production in *Capsicum* and the control of inorganic ions during water scarcity are suggested by its effects on water stress from exogenous melatonin (Kaya and Doganlar, 2019). Melatonin treatment can enhance proline accumulation, contributing to osmotic adjustment in specific crop species. In exogenous melatonin-treated rice, tomato, and maize, proline biosynthesis is enhanced through the upregulation of key enzymes such as Δ¹-pyrroline-5-carboxylate synthetase (P5CS), along with the suppression of protein-related processes involved in proline degradation (Siddiqui et al., 2019). Elevated levels of proline are the key player for osmotic adaptation and behave as molecular chaperones to protect proteins and membranes against denaturation due to drought stress and oxidative damage. Melatonin signaling-related regulation of proline is well correlated with increased cell turgor pressure, enhanced photosynthetic duration, and growth in seedlings under drought (Sharma and Zheng, 2019). Melatonin not only contributes towards proline synthesis but also helps retain soluble sugars (sucrose, glucose and fructose) as osmolytes and energy sources. These sugars aid in osmotic adjustment, stabilize cellular membranes, and protect macromolecules while supporting carbon metabolism and sustaining glycolytic activity under stress conditions (Ghouili et al., 2021). This sugar build-up related to melatonin treatment in tomato and cucumber plants in particular is responsible for the enhanced leaf hydration, longer leaf senescence and enhanced rehydration cycles after drought which are critical markers of metabolic homeostasis in both water-deficiency and drought-promoting periods. Mechanistically, melatonin regulates carbohydrate metabolism by modulated enzymes such as sucrose phosphate synthase and sucrose synthase, along with associated gene expression while also protecting photosynthetic machinery and influencing stress responsive genes (Zhao et al., 2021; Jahan et al., 2024). Melatonin also regulates crucial ion transporters that promote ion uptake in plant tissues, maintaining the ion balance. This regulatory process allows to neutralize cytotoxic stress and also promote turgor under drought stress (Shahmohammadi et al., 2024). For example, the application of exogenous melatonin to *Brassica rapa* was observed to enhance the uptake both in leaves and roots of potassium (K^+^) and zinc (Zn²^+^), and raise total soluble sugars and free amino acids, proteins, or photosynthetic pigments under drought conditions (Hasnain et al., 2023). Similar findings in rice and apple meristems further reveal that melatonin influences ion transporter-related genes, which are relevant for the balance of Na^+^/K^+^ at the cytoplasmic membrane site, such as the NHX and SOS family genes (Sun et al., 2023; Chen et al., 2021). Mechanistically, melatonin modulates the expression and activity of these transporters, thereby improving ion uptake, sequestration, and intracellular balance under stress. Besides, melatonin has a beneficial effect on membrane lipid profiles by alleviating lipid peroxidation while keeping unsaturated fatty acids necessary for the membrane’s fluidity at a sustained state under dehydration conditions. Similarly applied on kiwifruit seedlings (*Actinidia chinensis* var. deliciosa cv. Qinmei), it ameliorated damage induced by drought in response to dosage, reducing lipid peroxidation rates alongside membrane injury while promoting osmolyte accumulation. Under combined salt and drought stress, melatonin modulates plasma membrane H^+^-ATPase activity by enhancing proton pumping, which establishes the electrochemical gradient required for the efficient function of Na^+^/H^+^ antiporters. This regulation promotes K^+^ uptake and utilization that can also promote the K^+^/Na^+^ ratio, which could also result into greater resistance to salinity-induced stresses. These effects act synergistically with the previously observed osmotic regulation mediated by melatonin further improving plant stress tolerance (Khan et al., 2024). Furthermore, application led to significant decreases in electrolyte leakages while maintaining the cellular water balance in rice samples under dry conditions (Khan et al., 2024). At subcellular level, melatonin’s function contributes toward stabilizing organelles, primarily chloroplasts and mitochondria. Through its antioxidant properties and by boosting the storage of extra energy necessary for ATP production-vital for cellular carbon assimilation and redox balance. Melatonin mitigates oxidative and osmotic damage resulting from drought stress (Reiter et al., 2015; Sun et al., 2021). In summary, melatonin’s regulation of osmolyte accumulation by cellular homeostatic measures is a simple adaptation mechanism that allows physiological responses perceived during events to be linked with growth regulation processes.

## Microbe-mediated drought stress alleviation regulated by melatonin

It has recently been reported that melatonin not only directly improves plant drought tolerance, but also modulates the plant-microbe interactions that play a role in stress tolerance (Afridi et al., 2022; Zhang et al., 2015). Beneficial rhizosphere and endophytic microbial community members, such as plant growth-promoting rhizobacteria (PGPR) and mycorrhizal fungi, enhance plant performance under drought by increasing nutrient uptake, root development, and osmotic adjustment (Vurukonda et al., 2016). Melatonin-related effects on root architecture and root exudation behavior enhances recruitment and stabilization of drought-adaptive microbial communities. Melatonin− mediated alteration of the rhizosphere milieu enhances beneficial plant–microbe associations which promote water-use efficiency and stress resilience in drought conditions (Arnao and Hernández-Ruiz, 2019; Luo et al., 2024). Under drought stress, plants actively contribute to recruitment and enrichment of beneficial rhizospheric and endophytic microbial communities that produce different types of bioactive agents including osmolytes, phytohormones, antioxidants, and volatile organic compounds (Afridi et al., 2022). Microbial tolerance towards drought is associated with melatonin-dependent osmolyte accumulation and redox homeostasis in host plants. PGPR like *Bacillus*, *Pseudomonas* and *Azospirillum* further upregulate proline, soluble sugars and glycine betaine, which are osmoprotectants and stabilizing mechanisms in cells when dehydrated. These positive microbial effects are frequently synergized in plants treated with melatonin which results in improved osmotic balance, attenuation of membrane damage and increase of antioxidant enzymes. Melatonin also underpins microbial stress mitigation by regulating ROS signaling and enhancing cellular homeostasis that enables beneficial microbes to thrive under drought stress (Vurukonda et al., 2016; Imran et al., 2021). Under drought conditions, plant-associated microbiomes also modulate the expression of drought-responsive genes. As an example, strains of *Bacillus subtilis* have been found to promote the expression of important drought tolerance genes such as *DREB2* and *RD29* that lead to improved drought tolerance of rapeseed (*Brassica napus* L.) and *Arabidopsis* (Poudel et al., 2021). Emerging evidence suggests that melatonin further strengthens plant drought resilience through its interactions with beneficial microbes, including arbuscular mycorrhizal fungi (AMF) (Xia et al., 2022). Root-based melatonin application can alter rhizosphere microbial composition by stimulating bacterial populations involved in carbohydrate and carboxylate degradation in barley that have been identified as contributing to increased drought-resistant barley (Ye et al., 2022). From a functional perspective, the breakdown of these organic substrates increases the availability of low molecular weight carbon sources thereby supporting the proliferation and metabolic activity of beneficial microbes. This process may increase carbon availability, modulates rhizospheric pH, and promotes beneficial metabolites thereby improving nitrogen uptake, root efficiency and overall plant resilience under drought stress (Du et al., 2022). Endophytic bacteria and fungi from stress-adapted plants produce melatonin, which enhances host stress tolerance by strengthening antioxidant defenses. It also activates drought-responsive factors and genes, improving the plant’s ability to cope with drought stress (Jiao et al., 2016). The melatonin-microbe interaction occurs primarily through modulation of root exudates, where melatonin alters the release of sugars, amino acids, and organic acids that selectively recruit beneficial microorganism taxa (Aijaz et al., 2025). Additionally, melatonin acts as a signal molecule, influencing microbial processes such as quorum sensing, biofilm formation and stress responsive activity (Singh et al., 2025). Thus this interaction maintain plant immunity, microbial balance and prevents pathogen invasion under drought stress. However, the precise molecular signaling pathways remain insufficiently understood and warrant further investigation.

Moreover, certain bacteria associated with plants have the ability to produce melatonin, indicating a potential inter-kingdom mechanism by which microbes can improve plant resilience to stress. Both bacterial synthesized and externally applied melatonin have been shown to influence plant responses to abiotic stresses, including drought, salinity, UV radiation, and temperature extremes (Gamalero and Glick, 2025). This modulation occurs through the enhancement of antioxidant defenses, hormone signaling pathways, and changes in gene expression.

For example, Jofré et al. (2024) illustrated that the plant growth promoting bacterial strains *Pseudomonas* 42P4 and *Enterobacter* 64S1 increase endogenous melatonin levels in Arabidopsis thaliana subjected to drought stress. This increase results in enhanced growth, greater water deficit tolerance, and decreased oxidative damage. It was shown that when tomato plants were inoculated with these strains under drought conditions, there was an improvement in growth metrics such as chlorophyll content and photosynthetic efficiency alongside elevated endogenous melatonin levels (Liu et al., 2016). These treatments also resulted in higher proline accumulation and lower lipid peroxidation, contributing to improved drought resistance.

Moreover, Imran et al. (2023) investigated the joint application of melatonin with the PGPB *Lysinibacillus fusiformis* PLT16 in soybean plants facing water scarcity. This particular strain demonstrated several beneficial traits for plants, including the production of exopolysaccharides, siderophores, auxins, as well as exhibiting drought resistance. The combined use of melatonin and PLT16 led to improvements in hormonal balance, antioxidant capacity, physiological performance, and molecular responses, ultimately strengthening plant resilience under conditions of drought stress.

While these findings are promising, the pathways underlying melatonin-mediated interactions with plant-associated microbial communities under drought stress remain largely unexplored. Understanding this complex interplay represents an important frontier in plant stress biology. Studies may examine the signaling and communication pathways between melatonin and microbial population under drought events as a pathway toward future studies. Specifically, if we recognize that specific bacterial and fungal taxa are able to synergize with melatonin to enhance resistance to drought, it may provide new insights for enhancing crop resilience and sustainability in world crop production.

## Molecular mechanism of phytomelatonin in drought mitigation

**Figure 1:**
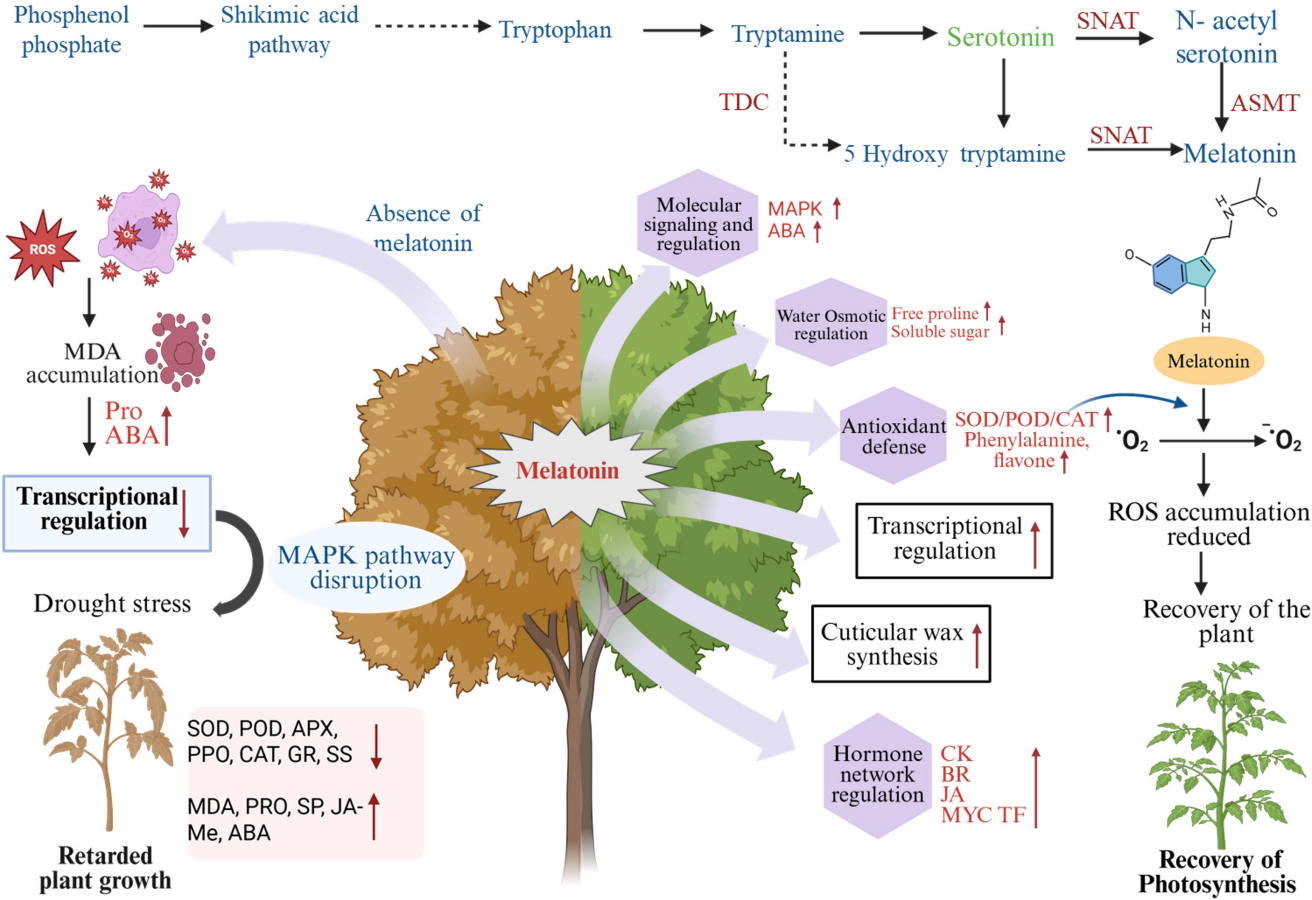
Biosynthesis of melatonin and its multi-functional capacity to modulate plant response to stress. The schematic depicts the melatonin biosynthetic pathway from phosphoenol phosphate through the shikimate pathway to tryptophan, where the latter is converted to tryptamine and serotonin, and melatonin through the action of important enzymes tryptophan decarboxylase (TDC), serotonin N-acetyltransferase (SNAT), and acetylserotonin O-methyltransferase (ASMT). Under stress, deficiency or low levels of melatonin result in excessive reactive oxygen species (ROS) accumulation, increased MDA, abscisic acid (ABA), impaired MAPK signal transduction, decreased transcriptional regulation, weakened antioxidant enzyme activities such as SOD, POD, APX, CAT, GR, SS, and retarded plant growth. Conversely, adequate melatonin balance can facilitate the increase of plant stress tolerance through the modulation of molecular signaling pathways (MAPK, ABA signaling) that are enhanced by the presence of melatonin, increasing the proline and soluble sugars for water osmotic control, the defense system of antioxidant (SOD, POD, CAT and secondary metabolites (phenylalanine/flavonoids), transcriptional regulation, cuticular wax biosynthesis, as well as the interaction between hormone network (CK, BR, JA and MYC transcription factors). Figure was created using BioRender.

Melatonin has been shown to boost plant resilience under drought conditions by promoting growth, photosynthesis, flowering, seed production, and aiding recovery following stress upon rehydration (Wang et al., 2022a). The action of melatonin on photosynthesis is well known in many plants, including tomato, maize, apple, and cotton (Ahmad et al., 2023; Khan et al., 2024). Melatonin increases chlorophyll accumulation and promotes better drought tolerance. This phenomenon is strongly related to high activity of important antioxidant enzymes including superoxide dismutase (SOD), ascorbate peroxidase (APX), catalase (CAT), and glutathione reductase (GR), which synergistically ameliorate the negative impacts of excessive ROS accumulation (Karaca and Cekic, 2019) (**Figure 1**). Importantly, pre-sowing melatonin in tomato cultivars like ‘Jinpeng No. 1’ boosts seedling vigor indices and diminishes drought-induced damage (Liu et al., 2015). Melatonin also improves stomatal conductance, photochemical efficiency, and synergies with antioxidant–ascorbate (AsA) systems, in order to reduce oxidative damage (Liu et al., 2015). Foliar melatonin application stimulated antioxidant enzyme activities that was also effective in inhibiting ROS accumulation and restoring redox homeostasis under high temperature (≥40 °C) and drought-induced combined stress conditions on field-grown tomato seedlings (Annadurai et al., 2023). Among *Platycrater arguta*, melatonin has been proposed to mitigate drought-induced oxidative stress by influencing the release of tryptophan metabolite and thereby H_2_O_2_ concentration and hormone signaling pathways (Zhang et al., 2025a). Further data indicate that melatonin promotes leaf cuticle production in drought-stressed tomato plants, preventing non-stomatal water loss and thereby delaying leaf wilting (Ding et al., 2018; Lee and Back, 2019). These findings reinforce the hypothesis of melatonin’s pivotal role in the evolutionary transition of aquatic plants into a terrestrial environment to undergo adaptation to land-based stresses. Genetically, melatonin appears to be implicated in drought responses as the upregulation of melatonin biosynthesis relevant genes such as *T5H* (*tryptamine 5-hydroxylase*), *HIOMT* (*hydroxyindole-O-methyltransferase*), *TDC* (*tryptophan decarboxylase*), and *SNAT* (*serotonin N-acetyltransferase*) is observed in apple under drought stress (Arnao and Hernández-Ruiz, 2015; Li et al., 2015; Wang L. et al., 2017). This rise implies that exposure to drought triggers melatonin biosynthesis, with melatonin as an important mediator of signaling intermediary. Endogenously regulated melatonin plays an essential role in the regulation of ROS homeostasis through rhythmic regulation of melatonin signaling by phytomelatonin signaling (**Figure 1**). The phytomelatonin receptor gene *PMTR1*, found in several plant species such as alfalfa, tobacco, maize and cassava, is known to act as a crucial signaling component promoting drought tolerance by contributing to stomatal closure (Wang et al., 2022b; Khan et al., 2023a). Homologs of *PMTR1* have been discovered in rice, indicating that these signaling functions are conserved across different species. These proteins are known to influence ion channels and stomatal behavior, which ultimately affects transpiration and drought resilience (Barman et al., 2023). In *Arabidopsis*, the perception of melatonin at the plasma membrane through PMTR1 leads to an interaction with the α-subunit of heterotrimeric G-proteins, which triggers the production of reactive oxygen species (ROS) and an influx of cytosolic Ca²^+^. These molecules serve as secondary messengers that facilitate stomatal closure and mediate stress responses (Wei et al., 2018). Moreover, signaling through PMTR1 activates MAPK cascades, notably the MKK4/5–MPK3/6 pathway. This activation is critical for managing transcriptional responses associated with plant immunity and resilience to stress (Khan et al., 2023a). PMTR1-mediated signaling orchestrates ROS and Ca²^+^ signaling networks to regulate downstream responses that enhance stress adaptation.

Notably, current transcriptional studies show that melatonin-mediated signaling quickly triggers massive gene expression involving ABA signaling, MAPK cascades, ROS-scavenging genes, and osmolyte biosynthesis pathways under short-term drought stress, reflecting an immediate stress-response phase (Li et al., 2026). Heterologous expression of *ZmPMTR1* in *Arabidopsis pmtr1* mutants restored their sensitivity to osmotic stress and reestablished phytomelatonin-induced stomatal closure (Wang et al., 2022b). Thermal infrared visualization revealed that maize *Zmpmtr1* mutants were found to be more drought sensitive than their wild-type peers exhibiting reduced leaf temperatures during drought stress, reflecting the importance of *ZmPMTR1* in maize adaptation to drought (Wang et al., 2022b; Khan et al., 2023a). On the other hand, long-term drought stress causes a more specific transcriptomic modification, with genes associated with osmo-protection and protein stabilization being operational while growth-associated mechanisms like cell wall organization, protein phosphorylation, and flavonoid biosynthesis are downregulated (Muthusamy et al., 2016). This indicates a shift toward adaptation rather than acute defense. Recent research has highlighted the role of genetic control strategies, such as enhancing *TaCOMT* expression in wheat and *MzSNAT5* in apples, in improving drought tolerance through melatonin (Yang et al., 2019). The application of exogenous phytomelatonin has also shown positive effects on crop growth, yield, and quality in species like *Moringa oleifera* and wheat (Wang L. et al., 2017; Cui et al., 2018; Sadak et al., 2020). In *Agrostis stolonifera*, treatment with melatonin led to the upregulation of genes responsive to drought stress (*JUB1* and *DREB2A*) while downregulating genes associated with chlorophyll degradation (*Chlase*, *pheophytinase*, and *Chl-peroxiredoxin*) (Sharma and Zheng, 2019). Likewise, genes involved in melatonin biosynthesis (*TDC1*, *SNAT1*, and *COMT*) exhibited significant activation (Ma et al., 2018). These molecular responses have recently been attributed to enhanced osmoregulation, augmented proline accumulation, enhanced antioxidant capacity, and a decrease in ROS-mediated oxidative stress (Gul et al., 2022). At the molecular level melatonin further modulates drought tolerance via metabolic pathways and transcription factors (Zhao et al., 2021).

Importantly, several transcription factors and downstream genes have been functionally linked to melatonin-mediated drought responses at a more direct regulatory level. Melatonin has been shown to upregulate DREB2A, which directly binds to dehydration-responsive elements in the promoters of stress-inducible genes such as *RD29A*, *RAB18*, and *KIN1*, thereby enhancing dehydration tolerance (Saify et al., 2026). Similarly, melatonin-induced WRKY transcription factors regulate antioxidant defense genes, including SOD, CAT, and APX, contributing to ROS detoxification (Imran et al., 2021; Hassan et al., 2022). Members of the NAC family, particularly JUB1, modulate downstream senescence- and stress-related genes, including repression of SAG12, thereby delaying drought-induced senescence (Wu et al., 2012). In addition, MYB and ERF transcription factors contribute to the activation of osmoprotective genes such as *P5CS* (proline biosynthesis) supporting osmotic adjustment under drought condition (Wang et al., 2025; He et al., 2025). Although these regulatory links are supported by transcriptomic and functional studies, future studies on chromatin-level validation is required to confirm direct TF–promoter interactions in melatonin signaling pathways. Beyond transcriptional regulation, melatonin reprograms key metabolic pathways to support stress adaptation. Short-term drought leads to rapid accumulation of compatible solutes such as proline, soluble sugars, and amino acids, aiding osmotic balance and ROS scavenging (Abbas et al., 2023). Melatonin enhances nitrate reduction and ammonium assimilation enzyme activities, thereby reshaping amino acid pools and soluble protein content, which indicates improved nitrogen redistribution and mobilization under drought conditions (Zhao et al., 2021; Khattak et al., 2023). Nevertheless, plants shift toward the formation of more stable osmo-protectants under prolonged periods of drought, such as trehalose, raffinose family oligosaccharides, and stress-induced secondary metabolites, which promote membrane strength and long-term intracellular protection (Kumawat et al., 2022). These responses are consistent with transcriptomic data indicating that melatonin-mediated modulation of genes for the TCA cycle, nitrogen, sucrose metabolism and amino acid biosynthesis is established. Furthermore, multidisciplinary omics research indicates that melatonin maintains metabolic homeostasis during long-term drought conditions and ensures quick activation during early stress by fine-tuning the time-dependent synchronization within transcriptomic and metabolomic responses (Wang et al., 2023a). These metabolic adjustments are closely coordinated with intracellular signaling networks. It was found that melatonin transcriptionally modulates protein kinases including MAPKs and calcium signaling kinases (CDPKs, CIPKs, and CRKs) which are known to be involved in drought stress signaling pathways (Arora et al., 2024). Calcium signaling molecules including calmodulin-like (CML) proteins have also been associated with melatonin-based drought tolerance. *CML44* up-regulated in tomato increases drought tolerance and melatonin activates CDPKs through Ca²^+^ signaling (Munir et al., 2016; Khan et al., 2023b). Melatonin application enhanced *CDPK1* and *CDPK2* expression in tomato leaves in the presence of increased H_2_O_2_ thus strengthening Ca²^+^ signaling and supporting a redox balance and adaptation to stress (Khan et al., 2023b). Melatonin pre-treatment has also been shown to enhance drought tolerance in tolerant (*Malus prunifolia*) and sensitive (*M. hupehensis*) apple species by preventing electrolyte leakage and maintaining chlorophyll stability, as well as enhancing water retention and photosynthetic activity. Apart from the ROS regulation, H_2_S has been identified as a key signaling molecule of melatonin-mediated drought responses. Exogenous melatonin improves H_2_S synthesis while regulating expression of the *LCD* (*L-cysteine desulfhydrase*) gene during normal and stress conditions. The stomatal closure mediated by the K^+^ channel-related genes (*AKT1*, *KC1*, *KCO1*, and *KAT1*) (enhanced drought tolerance in *Arabidopsis*) is due to the production of H_2_S (synthesized after melatonin treatment). Dysregulation of *lcd* and *des1* genes suppresses melatonin-associated drought tolerance, therefore confirming the indispensable contribution of H_2_S in this network of regulation (Wang et al., 2023c). Stable physiological gains after melatonin treatment have been studied in other plant species, such as Chinese hickory, tomato, fenugreek, kiwifruit, grapes, coffee- and in this context, the findings of the study show an increased photosynthetic efficiency, antioxidant activity, chlorophyll retention, turgor maintenance, and reduction in lipid peroxidation (Karaca and Cekic, 2019; Sharma et al., 2020; Ibrahim et al., 2020; Zamani et al., 2020; Altaf et al., 2022; Zhao and Hu, 2023). Melatonin may also enhance seed germination and premature seedling growth, contributing to its stress-resilience role (Bai et al., 2020). Foliar melatonin supplementation positively influenced growth characteristics, leaf area, photosynthetic performance in drought-stress in the crops *Moringa oleifera* and *Coffea arabica* (Sadak et al., 2020; Campos et al., 2019; Cherono et al., 2021). In addition to classical signaling pathways, emerging regulatory layers have also been identified. Emerging evidence indicates that melatonin may mediate drought tolerance via lncRNA–miRNA regulatory interactions, although knowledge of this mechanism is still limited (Ding et al., 2019). Similarly, application of rhizospheric melatonin promoted drought-resilient effects that were found to modulate oxidative and nitrogen metabolism (Antoniou et al., 2017). Melatonin modulate rhizosphere microbial communities by altering root exudate composition, thereby influencing microbial recruitment and activity (Bais et al., 2006). These changes can enrich PGPR that enhance drought tolerance through phytohormone production, exopolysaccharides, and ACC deaminase activity (Vurukonda et al., 2016). Collectively, melatonin–microbe interactions likely act synergistically to improve plant drought resilience. Melatonin systematically mitigated oxidative stress, increased antioxidant enzymatic activity, and boosted chlorophyll in tall fescue, fenugreek, and soybean; as a result, their drought resistance was significantly enhanced (Alam et al., 2018; Zamani et al., 2020; Imran et al., 2021).

Transgenic studies also emphasize melatonin as a promising crop enhancer. Ectopic expression of the human melatonin synthase gene *HIOMT* in apples significantly removed long-term drought stress by increasing growth, photosynthesis, chlorophyll content and nitrogen uptake efficiency (Liang et al., 2023). Transgenic lines exhibit higher activity of nitrogen metabolic enzymes and strong induction of the nitrogen uptake and assimilation genes compared to wild-type plants. The same is true for expression of *MmCYP1A1*, a cytochrome P450 monooxygenase active in melatonin metabolism, which further enhances melatonin and 6-hydroxymelatonin levels in apple calli and *Arabidopsis*, favoring tolerance to drought and osmotic stress (Wang et al., 2023b). These plant species showed lower ROS accumulation, lower MDA levels, greater root growth and expression of stress response genes, and improved stomatal regulation (Tan et al., 2021; Wang et al., 2023b). Melatonin application downregulates relative expression of *SlNCED3* and *SlDREB3* genes in tomato plants under drought stress. SlNCED3 encodes 9-cis-epoxycarotenoid dioxygenase, a rate-limiting enzyme in abscisic acid (ABA) biosynthesis, and its reduced expression under melatonin treatment indicates a suppression of excessive ABA accumulation during drought (Shaffique et al., 2024). ABA is essential for initiating stress responses such as stomatal closure, prolonged or excessive ABA signaling often leads to growth inhibition and premature senescence. Melatonin-mediated downregulation of *SlNCED3* therefore, suggests a fine-tuning mechanism whereby stress defense is maintained without compromising growth and metabolic activity. The downregulation of SlDREB3, a member of the DREB transcription factor family, further highlights melatonin’s role in modulating stress signaling intensity (Shaffique et al., 2024). DREB transcription factors are central regulators of drought-responsive genes involved in osmotic adjustment, ROS detoxification, and stress- induced metabolic reprogramming (Agarwal et al., 2017). By modulating SIDREB3 expression, melatonin likely prevents excessive activation of stress signaling pathways, thereby enhancing stress tolerance while maintaining cellular homeostasis and photosynthetic efficiency (Shaffique et al., 2024). These findings indicate that melatonin functions not merely as a stress signal amplifier but as a regulatory buffer that optimizes drought responses through hormonal and transcriptional reprogramming. By suppressing excessive ABA biosynthesis (SlNCED3) and fine-tuning drought-responsive transcription (SlDREB3), melatonin enables tomato plants to achieve a balanced response characterized by improved water-use efficiency, reduced oxidative damage, sustained growth, and enhanced drought resilience (Shaffique et al., 2024). Transcriptomic profiling of cotton showed that melatonin application under drought stress modulated genes related to thiamine metabolism, circadian rhythm regulation, and taurine and hypotaurine metabolism, underscoring melatonin’s extensive regulatory potential (Zhong et al., 2025). Additionally, comparative omics studies show that recovery from long-term drought requires a more gradual metabolic adjustment and sustained antioxidant activity while in short-term drought melatonin improves recovery-time for transcriptomic modification after rehydration by quickly rebuilding photosynthesis-associated genes (Du et al., 2024). Likewise, the exogenous melatonin provided as a biostimulant in *Zinnia elegans*, with benefits in drought tolerance, osmotic adjustment, membrane stabilization and stomatal regulation, and ultimately improved water balance and photosynthesis performance (Coêlho et al., 2025).

## Crosstalk between melatonin and phytohormones to mediate drought tolerance in plants

**Figure 2:**
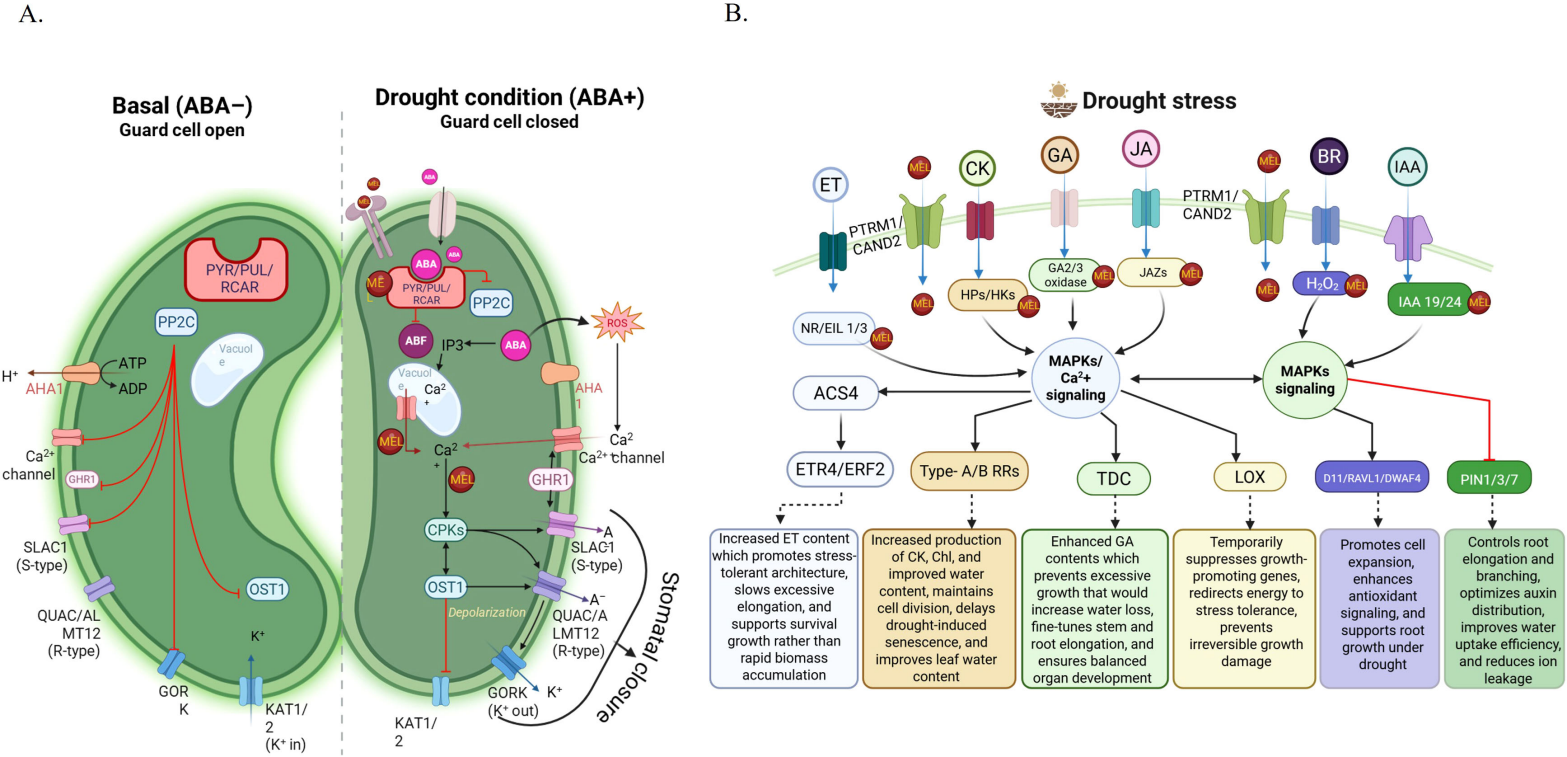
**(A)** Proposed model of melatonin–abscisic acid (ABA) crosstalk regulating guard cell signaling and stomatal movement under stress conditions. The diagram contrasts a low-melatonin/ABA-inactive state (left) with a melatonin- and ABA-activated state (right). Under stress, melatonin (MEL) enhances ABA perception by the PYR/PYL/RCAR receptor complex, leading to inhibition of PP2C phosphatases and activation of downstream ABA signaling. This triggers ABF-dependent transcriptional responses, IP_3_-mediated Ca²^+^ release from the vacuole, and elevation of cytosolic Ca²^+^ levels. Increased Ca²^+^ activates CPKs (Calcium-dependent protein kinases) and OST1 (OPEN STOMATA 1), promoting phosphorylation of ion channels and transporters, including SLAC1 (S-type anion channel), QUAC1/ALMT12 (R-type anion channel), and GORK (K^+^ outward channel), while inhibiting KAT1/2 (K^+^ inward channels); **(B)** Melatonin-induced hormonal crosstalk and signaling pathways under drought stress. Drought stress triggers complex interactions between MEL and multiple phytohormones, including ethylene (ET), cytokinins (CK), gibberellins (GA), jasmonic acid (JA), brassinosteroids (BR), and auxin (IAA). Melatonin modulates hormone perception and downstream components such as HPs/HKs, GA2/3 oxidase, JAZs, H_2_O_2_, and IAA19/24, leading to the activation of MAPKs and Ca²^+^ signaling pathways. These signaling cascades regulate key transcriptional and metabolic nodes (e.g., ACS4, ETR4/ERF2, type-A/B RRs, TDC, LOX, D11/RAVL1/DWAF4, and PIN1/3/7), resulting in coordinated physiological outcomes. Figure was created using BioRender.

Phytohormones are a key component of the ability of plants to perceive, respond to, and endure drought stress. Among them, melatonin has developed as one of the major molecules of regulation. Melatonin is a central player in a complex hormonal system rather than acting alone, coordinating with several plant growth regulators to adapt drought-stress responses (**Table 2**). Recent studies have shown extensive crosstalk of melatonin with nearly all of the main classes of plant hormones (Khan et al., 2022; Ali et al., 2024). These interactions include classical hormones like auxin, cytokinins, gibberellins (GAs), and abscisic acid (ABA), and less typically focused regulatory agents, such as salicylic acid (SA), jasmonic acid (JA), brassinosteroids (BRs), polyamines, and strigolactones (Khan et al., 2022; Ali et al., 2024) (**Figure 2**). Due to similarities in their structure, melatonin-hormone interactions were initially focused on auxin (IAA). The interaction between auxin signaling and other phytohormones is essential for effective stress responses (Ali et al., 2025). Morphological evidence strongly supports the auxin-like role of melatonin, particularly under drought conditions, where it enhances shoot elongation, root growth, and the development of lateral and adventitious roots. In *Moringa oleifera* L. melatonin was shown to induce enhanced endogenous IAA levels under water-limited conditions with foliar addition melatonin in varying concentrations of 100 to 150 mM (Sadak et al., 2020). Lower concentrations of melatonin (i.e., <10 μM) have been reported to enhance seed germination and stimulate lateral root formation in cucumber plants under cold and drought stress conditions (Zhang et al., 2013; Simlat et al., 2018). These concentration dependent effects highlight the role of melatonin in modulating auxin signaling and development, emphasizing that physiological outcomes depend strongly on dosage (Wen et al., 2016; Zhang et al., 2022). In addition, melatonin-ABA interaction is well documented in drought stress (Liang et al., 2023). ABA is a stress hormone, an endogenous metabolite which rapidly builds up in response to drought stress and exhibits a strong association with oxidative stress and stomatal closure. Melatonin regulates ABA-associated signaling genes under drought stress, including *SnRK2* and *RCAR*/*PYR*/*PYL* (Dzinyela et al., 2024). In apple, melatonin pretreatment enhances drought tolerance by promoting antioxidant defenses and scavenging H_2_O_2_. Overall, melatonin improves drought resilience through two key mechanisms: modulation of ABA homeostasis (both synthesis and degradation) and detoxification of drought-induced ROS (Arnao and Hernández-Ruiz, 2015; Kanwar et al., 2018). Exogenous melatonin application further enhances drought tolerance and delays leaf senescence, often associated with reduced ABA levels and downregulation of ABA-responsive genes (Kanwar et al., 2018; Zhang et al., 2022). Although drought stress typically upregulates ABA biosynthesis genes, melatonin treatment suppresses their expression under stress conditions (Dzinyela et al., 2024). In apple, melatonin alters ABA accumulation and improves drought priming-induced tolerance (Banerjee et al., 2023; Dzinyela et al., 2024). Similarly, genetic studies in rice show that mutations in ABA signaling components such as OsABI5 and OsSGT1 enhance melatonin-mediated drought tolerance (Dzinyela et al., 2024). Furthermore, melatonin delays drought-induced leaf senescence by inhibiting ABF-mediated ABA biosynthesis, providing insight into the molecular interplay between melatonin and ABA in stress regulation (Zhang et al., 2022). Furthermore, it has also been demonstrated that melatonin downregulates drought-mediated *NCED1* (*9-cis-epoxycarotenoid dioxygenase*) gene, one of the important ABA biosynthetic genes (Rehaman et al., 2021). Simultaneously, melatonin increases the expression of ABA catabolic genes such as *ABA8ox1* and *ABA8ox3*, which reduces ABA levels and increases stomatal reopening in maize during drought (Li et al., 2021).

**Table 2:** Melatonin crosstalk with plant hormones to improve drought stress tolerance in plants in last decade.

Plant species	Involved phytohormone	Mechanism	References
*Malus hupehensis*; *Malus prunifolia*	Abscisic acid	• Downregulates the gene responsible for ABA synthesis (*MdNECD3*) • Upregulates genes involved in ABA degradation(*MdCYP707A1*, *MdCYP707A2*) • Enhances the expression of melatonin biosynthetic genes (*MdTDC1*, *MdAANAT2*, *MdT5H4*, and *MdASMT1*), leading to the opening of stomata	Li et al., 2015
*Zea mays*	Abscisic acid	• Inhibits upregulation of ABA synthesis-related gene (*NECD1*) • Promotes upregulation of ABA catabolicgenes (*ABA8ox1* and *ABA8ox3*) • Increases endogenous melatonin levels, thus opening stomata	Li et al., 2021
*Zea mays*	Abscisic acid	• Exogenous melatonin treatment leads to downregulation of ABA in drought stress. This mechanism mediated the Ca2+ signal transduction-related genes, and induction of theseries of downstream TFs such as AP2/ERF-ERF, WRKY, MYB, NAC, bHLH, and bZIP	Zhao et al., 2021
*Arabidopsis thaliana*	Abscisic acid	• Redox homeostasis via ROS scavengingupregulation of SOD, CAT, APX; ABA signaling modulation; regulation of stress-responsive TFs (WRKY, NAC)	Arnao and Hernández-Ruiz, 2014; Zhao et al., 2019
*Medicago sativa*	Gibberelic acid and Abscisic acid	• Upregulates ASMT (MsG0480020076.01) expression under drought stress	Zhang et al., 2025b
*Vitis vinifera*		• Regulation of stomatal conductance; ROS detoxification; chloroplast protection	Gao et al., 2022
*Citrullus lanatus*	Abscisic acid	• Downregulation of ABA	Dzinyela et al., 2024
*Malus domestica*	Abscisic acid	• Inhibited biosynthesis of ABA	Xu et al., 2019
*Agrostis stolonifera*	Cytokinin	• Melatonin crosstalk with cytokinin leads to upregulation of cytokinin (signaling, and synthesis genes)	Ma et al., 2018
*Citrullus lanatus*	Ethylene	• Melatonin crosstalk with ethylene stimulates ethylene metabolism	Kaya and Ugurlar, 2023
*Solanum lycopersicum*	Ethylene	• Melatonin crosstalk with ethylene improved ethylene gene expression	Colombage et al., 2023
*Brassica napus*	Gibberelins	• Melatonin Synergistic effect with GA3 leads to drought tolerance	Khan et al., 2020
*Oryza sativa*	_	• Activated the expression of melatonin synthesis gene (*TDC*)	Wang et al., 2024
*Moringa oleifera*	Auxin	• Melatonin induces level of endogenous IAA	Tiwari et al., 2021
*Triticum aestivum*	Jasmonic acid	• Melatonin crosstalk with Jasmonic acid regulated molecular transcripts • including JA-JIM-domain proteins	Shreya et al., 2022
*Zea mays*	Salicylic acid	• Improved SA biosynthesis	Moustafa-Farag et al., 2020
*Brassica napus*	Salicylic acid	• Melatonin induces level of endogenous SA and leads to drought tolerance	Rafique et al., 2024
*Hibiscus syriacus*	Salicylic acid	• Upregulates antioxidant enzyme activity • Improved photosynthetic system • Upregulated drought-related response genes	Yan et al., 2024
*Solanum lycopersicum*	Salicylic acid	• Melatonin cross-talks with salicylic acid regulated the oxidative-nitrosative processes • Upregulates methylglyoxal metabolism	Kaya and Ugurlar, 2023
*Arabidopsis thaliana*	Brassinosteroids	• Phytomelatonin and brassinosteroids promote stomatal reopening by either ABA degradation or suppressing its biosynthesis through the downregulation of NCED genes.	Waseem et al., 2025

Comparative regulation pattern has been noted in apple where exogenous melatonin has downregulated *MdNCED3* but increased the expression of ABA degradation genes (*MdCYP707A1* and *MdCYP707A2*) in tolerant (*Malus prunifolia*) and sensitive (*M. hupehensis*) genotypes (Li et al., 2015). Interestingly, drought stress produced the expression of melatonin biosynthetic genes (*MdTDC1*, *MdAANAT2*, *MdT5H4*, and *MdASMT1*), therefore melatonin biosynthesis is also stimulated during water deficit (Ali et al., 2023). Although ABA-dependent stomatal closure should be controlled to reduce the water losses during a drought, a long-term closure is negatively associated with CO_2_ uptake, limiting photosynthesis and biomass. The rapid reopening of stomata after stress alleviation is consequently important to plant recovery. Increasingly, it is being demonstrated that melatonin and brassinosteroids act to antagonize ABA-mediated stomatal closure, either by depressing ABA biosynthesis or promoting its degradation. This attenuates the H_2_O_2_ buildup induced by ABA in guard cells, decreases Ca²^+^ signaling, and promotes activation of KAT1 or other inward-rectifying K^+^ channels leading to stomatal re-opening (Waseem et al., 2025). Under drought stress, melatonin modulates phytohormone homeostasis in maize by regulating the biosynthesis of gibberellic acid (GA_3_) and indole-3-acetic acid (IAA), while promoting the catabolism of abscisic acid (ABA) (Ahmad et al., 2023). The plant hormones ABA and GA_3_ have antagonistic effects on each other under drought stress which results in the elevation of ABA, suppressing the level of plant growth hormones GA_3_ and IAA. However, the exogenous application of melatonin significantly increased the GA_3_ and IAA levels in maize leaves (Ahmad et al., 2022). Melatonin has been reported to enhance GA biosynthesis and signaling in drought conditions, opposite to its suppressive effect on ABA accumulation (Talaat, 2023). Rapeseed had significantly better plant growth, yield, and seed quality following combined priming with GA_3_ and melatonin under drought stress. In cucumber (*Cucumis sativus*), melatonin stimulates lateral root formation and root hydraulic conductivity while delaying drought-induced leaf senescence through regulation of auxin and senescence-associated genes (Wang et al., 2016). Under drought stress, exogenous melatonin treatment upsurges GA2 oxidase and GA3 oxidase enzymes, hence inducing GA production (Khan et al., 2020; Liu et al., 2018). Compared to non-primed plants under drought stress, GA3 and melatonin-primed rapeseed plants displayed better morphological features, yield and yield components, and qualitative seed qualities (Dzinyela et al., 2024).

Melatonin interacts with other stress-relating hormones such as JA and SA. Endogenous ABA and JA levels were found to be increased with melatonin treatment in the maize and these effects were reinforced by increased expression of genes for hormone biosynthesis and stress-responsive responses in maize, which collectively enhanced drought tolerance (Ahmad et al., 2023). Altered expression of calmodulin-like (CML) proteins further connects melatonin to SA and ABA signaling in *Arabidopsis*, including CML8, which positively regulates SA response and overexpression of CML20 reducing ABA signaling and a decrease in drought tolerance (Zhu et al., 2017; Wu et al., 2017). Melatonin and ABA also regulate wax metabolism by modifying alkane biosynthesis (C29 and C31) and wax crystal production contributing to minimized non-stomatal water loss during drought stress and is crucial for watermelon (Li et al., 2020).

Melatonin also regulates auxin biosynthesis pathways and stimulates cytokinin production by upregulating genes encoding histidine kinases, response regulators, and phosphotransfer proteins, all of which lead to optimal drought tolerance (Wei et al., 2022; Sharma and Zheng, 2019). Melatonin enhances CK production by upregulating genes involved in CK signaling, including histidine kinases, histidine phosphotransfer proteins, and response regulators (Type-A and Type-B RRs), thereby improving drought tolerance (Kanwar et al., 2018; Dzinyela et al., 2024).

Together, melatonin and CKs improve key physiological traits such as chlorophyll content, photochemical efficiency, and relative water content in both drought-stressed wild-type and isopentenyl transferase-overexpressing transgenic creeping bentgrass (*Agrostis capillaris*), ultimately enhancing drought resilience (Sati et al., 2023; Dzinyela et al., 2024).

Brassinosteroid biosynthesis is another hormonal pathway with melatonin’s positive regulation. Melatonin induces multiple BR biosynthetic genes resulting in optimal stress tolerance (Moustafa-Farag et al., 2020; Hwang and Back, 2018).

Recent evidence indicates that melatonin significantly increases endogenous BR content by transcriptionally activating key BR biosynthesis and regulatory genes, including *CY750A1*, *CYP707A5, CYP707A7*, *CYP87A3*, and *CYP90D2* (Fu et al., 2022). These cytochrome P450 monooxygenases are involved in multiple steps of BR biosynthesis and hormone homeostasis, and their coordinated upregulation by melatonin leads to elevated BR accumulation under drought and cold stress. Enhanced BR levels contribute to improved cell expansion, vascular development, and maintenance of membrane integrity, which are critical for sustaining growth under stress conditions. Melatonin-mediated BR accumulation reinforces stress tolerance by modulating downstream BR signaling pathways, including the activation of BR-responsive transcription factors such as BZR1 (Brassinazole Resistant 1) and BES1 (Brassinosteroid Insensitive 1-EMS-Suppressor 1) (Xiong et al., 2019). This activation enhances the expression of stress-responsive genes involved in antioxidant defense, osmotic adjustment, and photosynthetic protection. Increased BR signaling under melatonin treatment has been associated with reduced ROS accumulation, enhanced activities of antioxidant enzymes (SOD, CAT, APX), and stabilization of chloroplast ultrastructure, thereby protecting photosynthetic efficiency during drought and cold stress (Fu et al., 2022; Arnao and Hernández-Ruiz, 2019). Similar upregulation of BR biosynthesis genes has been reported in Chinese hickory, rapeseed, and *Davidia involucrata*, where upregulation of endogenous BR levels promoted stress resilience (Sharma et al., 2020; Tan et al., 2019; Liu et al., 2021). Melatonin shows a great deal of crosstalk with JA signaling. In maize, transcriptomic analyses showed that melatonin treatment affected the gene expression of hundreds related to JA biosynthesis and signaling during drought-induced stress (Zhao et al., 2021). Exogenous melatonin increased JA accumulation in soybean, rice, and wheat, which decreased water loss and improved antioxidant defense to counteract stress response (Imran et al., 2021; Li et al., 2022a; Wang et al., 2022b). In rice, melatonin restored decreases in JA as a result of drought, and then activated OsSGT1, which is a major regulator of gene expression of antioxidant genes (Li et al., 2022b). Melatonin with methyl jasmonate treatments also enhanced osmolyte uptake, antioxidant potential, sulfur absorption capacity, and photosynthetic efficacy, especially at thermal stress conditions due to synergistic effect (Sehar et al., 2023). Yet, antagonistic interactions have been described as well. In salinity-stressed rapeseed, melatonin suppressed JA biosynthesis by upregulating *HPL1*, downregulating *AOC*, and boosting JAZ repressor proteins, thus inhibiting JA signaling (Xu et al., 2024). These contradictory results imply melatonin–JA interactions are context-dependent, and would warrant further studies.

A functional association between melatonin and SA signaling has been found. Furthermore, the expression of the drought-related response genes HsNCED and HsDBF1 in *Hibiscus syriacus* were upregulated by melatonin and SA (Dzinyela et al., 2024). There has been reported an accelerated SA accumulation in soybean after foliar and root-applied melatonin under drought stress, which reflects a synergistic role for melatonin expression during stress (Imran et al., 2021). Melatonin affects nitric oxide signaling via two interconnected mechanisms involving nitrate reductase (NR) and S-nitrosoglutathione reductase (GSNOR). In tomato, melatonin treatment promoted endogenous NO production by potentiating NR activity and inhibiting GSNOR activity, resulting in enhanced drought tolerance (Pardo-Hernández et al., 2020; Martínez-Lorente et al., 2022; Zhang et al., 2022). Moreover, melatonin-induced NO synergism effectively stimulated the transcription factors of drought-dependent (bZIP, WRKY27, MYB174) which enhanced soybean plant cell tolerance during drought (Imran et al., 2021; Samanta and Roychoudhury, 2023). Taken together, this study highlights that melatonin serves as a principal regulator of hormonal networks during drought stress. Besides, polyamines play a critical role in mitigating the adverse effects of various abiotic stresses, including drought (Napieraj et al., 2023). These molecules stabilize cellular membranes, scavenge ROS, regulate ion homeostasis, and modulate gene expression associated with stress responses. By maintaining osmotic balance and enhancing antioxidant defense, polyamines help preserve cellular turgor and metabolic activity under water-limited conditions, thereby promoting plant survival and growth. Furthermore, polyamines interact with phytohormone signaling pathways, such as ABA and ethylene, to fine-tune stomatal closure and transpiration efficiency during drought stress, highlighting their integrative role in stress adaptation (Altaf et al., 2023; Zhao et al., 2022). A study reported that drought stress significantly increased ethylene-related gene expression in tomato, whereas melatonin-pretreated plants showed expression levels comparable to or lower than unstressed controls (Colombage et al., 2023). Additionally, melatonin modulates ethylene perception and signaling; it enhances fruit ripening and quality by upregulating *ACS4* and other ethylene signaling genes, including *EIL1*, *EIL3*, *NR*, *ETR4*, and *ERF2*, thereby increasing ethylene production in tomato (Dzinyela et al., 2024).

In parallel, increasing attention has been directed toward strigolactones (SLs), a class of multifunctional carotenoid-derived plant hormones (Samynathan et al., 2024; Thula et al., 2023). Drought stress reduces the production of SL in non-mycorrhizal plants, resulting in the decline in germination stimulatory activity. This effect is more pronounced in tomato than in lettuce, indicating a higher drought sensitivity of SL biosynthesis (Ruiz-Lozano et al., 2016). In contrast, arbuscular mycorrhizal plants showed increased SL production under severe drought conditions (Ruiz-Lozano et al., 2016). Under drought conditions, exogenous application of strigolactones significantly decreased stomatal aperture, oxidative damage, and electrolyte leakage, while simultaneously enhancing plant growth and development, antioxidant enzyme activities, relative water content, chlorophyll fluorescence parameters, chlorophyll content, and gas exchange traits in *Vitis vinifera* (Min et al., 2019). Consistently, strigolactone treatment significantly increased stomatal sensitivity in tomato plants exposed to drought stress (Visentin et al., 2025). Plants also harbor potent antioxidant molecules, such as dopamine and melatonin, which have garnered considerable attention for their roles in enhancing stress tolerance. These compounds are essential regulators of diverse biochemical and physiological processes involved in plant growth and development. Under drought stress, both dopamine and melatonin significantly enhance nitrogen uptake and metabolism (Du et al., 2022). In *Malus hupehensis*, the exogenous application of dopamine reduced the burst of H_2_O_2_, a ROS whose level rapidly increases during stress condition (Du et al., 2022). It is also demonstrated that long-term application of dopamine to the soil retards the drought induced leaf senescence (Lian et al., 2018). Melatonin and dopamine have been found to play vital role in reduction of ROS and increase the level of free proline and soluble sugar in leaves of *Malus hupehensis* under drought stress (Du et al., 2022). They also increased the levels of antioxidants SOD, CAT, APX simultaneously mitigating the decrease in the accumulation of N15 isotope in leaves, stem and root under stress conditions (Du et al., 2022). These findings suggest that the interactive roles of melatonin, polyamines, and strigolactones in drought stress responses warrant further investigation to fully elucidate their coordinated regulatory mechanisms.

## Prospects of melatonin-mediated genetic and metabolic engineering in improving plant drought tolerance

After melatonin in plants was discovered (Dubbels et al., 1995; Hattori et al., 1995), significant interest was diverted into the study of melatonin biosynthetic pathway. Consequently, research on phytomelatonin has increasingly focused on elucidating its biosynthesis in plants, with studies employing both molecular and enzymatic approaches. Such studies have also identified that plants have a significantly different biosynthetic pathway to melatonin synthesis from that in animals. The first reactions are distinguished due to the hierarchical order of the first reactions. In plants, tryptophan is decarboxylated first to form tryptamine by tryptophan decarboxylase (TDC), the first intermediate to be hydroxylated at the 5-position of the indole ring by tryptamine 5-hydroxylase (T5H), with subsequent production of 5-hydroxytryptamine (serotonin) (Arnao and Hernández-Ruiz, 2018; Xu et al., 2024; Yolcu et al., 2025). This sequence is at variance with that commonly observed in mammals. Furthermore, the final steps in the biosynthesis of melatonin in plants are different than those in animals. The gap in plant and animal melatonin biosynthetic pathways underscores the divergence in reaction order, enzyme type, and metabolic intermediates, with distinct evolutionary roots of species generating melatonin from the two kingdoms. While this progress has been made, some issues with the pathway are not yet being resolved. However, the regulation of melatonin biosynthetic pathways may differ among plant species and genotypes, suggesting that the contribution of individual enzymes and regulatory networks to drought tolerance is not universally conserved (Liu et al., 2022).

Despite a general consensus on the tryptophan-to-serotonin biosynthetic route, uncertainties persist regarding the specific enzymatic steps responsible for serotonin-to-melatonin conversion (Arnao and Hernández-Ruiz, 2015; Back et al., 2016). Under physiological condition, the kinetic pathway by direct action of N-acetylserotonin to melatonin (via SNAT followed by ASMT or COMT) is physiologically preferred (Xie et al., 2022). Alternatively, a potential route mediated by O-methylation (serotonin → 5-methoxytryptamine → melatonin) has also been detected in many plants, potentially further under specific taxa or abiotic stress conditions (Back et al., 2016; Tan et al., 2016; Mannino et al., 2021). In the absence of stressful environment, moderately high melatonin levels seem to promote normal plant growth and development, whilst excessive levels may be inhibitory (Arnao and Hernández-Ruiz, 2014). In contrast, environmental stress usually requires a massive amplification of melatonin production, as a way of averting the plant damage (Arnao and Hernández-Ruiz, 2015; Back et al., 2016; Tan et al., 2016). Stress-induced changes in the expression and activity of various enzyme isoforms involved in melatonin biosynthesis are believed to be driving this adaptive response (Back et al., 2016). The regulation of plants’ production of melatonin in the ambient environment (e.g., light intensity and spectral quality) has been proposed and is currently not entirely understood. Variations in the expression of genes encoding melatonin biosynthetic enzymes with varying blue and far-red light and seasonal changes indicate a complex regulatory network. Abiotic stresses strongly potentiate the expression of serotonin *N-acetyltransferase* (*SNAT*) and *N-acetylserotonin methyltransferase* (*ASMT*)/*COMT* genes, but play an especially salient role in the pathophysiology related to ecological factors in plant species with respect to environmental factors by boosting its expression (Arnao and Hernández-Ruiz, 2015; Back et al., 2016). At least some of these responses are mediated via mitogen activated protein kinase (MAPK) signaling pathways.

Overexpression or knockout of melatonin biosynthesis genes has been shown to directly modulate endogenous melatonin levels, thereby influencing plant stress tolerance (**Table 3**). Melatonin’s biological importance is also underscored by its remarkable antioxidant ability since it is more effective than many other antioxidants at scavenging reactive oxygen and nitrogen species (Cai et al., 2025). Increased levels of SNAT and ASMT/COMT transcript and proteins in response to stress further bolster this protective role. In plants, critical melatonin biosynthetic enzymes (e.g., SNAT and ASMT) are localized in chloroplasts and mitochondria and are the source of *in situ* melatonin synthesis in the chloroplasts and mitochondria (Byeon et al., 2014; Back et al., 2016). The high metabolic activity of the compartments together with the plentiful acetyl-CoA concentration here present favorable conditions for acetylation reaction crucial for melatonin generation (Tan et al., 2016). Additionally, chloroplasts and mitochondria are major sources of ROS, so localized melatonin synthesis is an effective tool for the adaptive response to oxidative stress.

**Table 3:** Genetic regulation of melatonin biosynthesis as a strategy to improve plant stress tolerance.

Plant species	Gene name	Function	References
*Nicotiana tabacum* L.	*NtCOMT1*	Overexpression of *NtCOMT1* promotes drought resistance by increasing melatonincontent	Yao et al., 2022
*Citrullus lanatus* L.	*ClCOMT1*	Overexpression of *ClCOMT1* enhances transgenic *Arabidopsis* tolerance against cold,drought, and NaCl	Chang et al., 2021
*Triticum aestivum* L.	*TaCOMT*	Overexpression of the wheat *TaCOMT* gene enhances drought tolerance and increasesthe content of melatonin in transgenic *Arabidopsis*	Yang et al., 2019
	*ASMT-like*	Drought and heat stress induce ASMT like gens to elevate melatonin and activate stress-responsive transcription factors. Where reduced expression correlates with stress sensitivity and overexpression enhances tolerance to both stresses	Li et al., 2024
*Vitis vinifera* L.	*VvASMT1*	Ectopic overexpression of *VvASMT1* in *Nicotiana benthamiana* significantly enhances melatonin production and increased tolerance to salt and osmotic stresses	Yu et al., 2022
*Arabidopsis thaliana*	*SNAT*	SNAT-driven melatonin biosynthesis during drought and salinity stress enhances antioxidant defence and redox homeostasis. Mutant of this gene accumulate excessive ROS and show severe stress sensitivity, whereas the overexpression of this SNAT gene elevates the melatonin levels and confers strong tolerance to water deficit and salt stress	Arnao and Hernández-Ruiz, 2015
*Oryza sativa*	*SNAT*	Drought stress upregulates *SNAT* to increase melatonin and improve antioxidant capacity, where low melatonin mutants show growth inhibition and overexpression enhances drought tolerance and yield stability	Hwang and Back, 2018
*Setaria italica*	*SiSNAT*	Heat and drought stress activate *SiSNAT* to increase melatonin and protect photosynthesis, where reduced expression lowers stress tolerance and overexpression enhances survival under combined stresses	Meng et al., 2025; Ren et al., 2025
*Cicer arietinium*	*CaSNAT*	Under water deficit, Ca *SNAT*- mediated melatonin biosynthesis supports redox balance and growth, with reduced expression causing drought sensitivity and overexpression enhancing stress resilience	Karimian et al., 2025
*Gossypium hirsutum*	*GhSNAT*	Drought and salinity stress induce *GhSNAT* expression to enhance melatonin accumulation and cellular protection, where knockdown plants show growth inhibition and overexpression improves stress endurance	Chen et al., 2021; Bai et al., 2020
*Zea mays*	*ZmSNAT*	Under drought stress, *ZmSNAT*-mediated melatonin synthesis enhances osmotic adjustment and ROS detoxification, where mutants display reduced drought tolerance and overexpression improves water-use efficiency and plant survival	Guo et al., 2024
*Camellia sinensis*	*CsSNAT*	Drought and heat stress induce *CsSNAT* expression to enhance melatonin-mediated redox balance with reduced expression increasing stress injury and overexpression improving tolerance	Li et al., 2019

Metabolic engineering advances offer hope for improving melatonin biosynthesis by increasing precursor availability, modulating pathway enzymes’ expressions and optimizing cofactor usage (Zhang et al., 2021). For better catalytic efficiency of enzymes, detailed knowledge of the assembly of enzymes and how the reactions occur is essential for obtaining important residues to mutagenize. Current computational tools (such as DeepMind’s AlphaFold), for instance, have tremendous potential in guiding enzyme engineering. Genetic modifications have been considered one of the promising strategies for the analysis of melatonin as an environmental and physiological regulator of plant growth and stress coping (Jumper et al., 2021; Li et al., 2026). Agrobacterium-mediated transformation has recently successfully been used to modulate melatonin biosynthetic genes (Lee and Back, 2017). Elevation of endogenous melatonin levels through overexpression of these genes has been demonstrated to be effective for stress tolerance in various plant species including tomato, apple, rice, and wheat, therefore it represents a very broad potential for melatonin-based technology in enhancing crop resilience (Liu et al., 2019; Gao et al., 2024; Lee and Back, 2024; Shamloo-Dashtpagerdi and Aliakbari, 2025).

## Challenges and perspectives of melatonin applications for drought mitigation

The concept of employing melatonin to halt drought damage in crops has been rising in popularity in the last few years. Although melatonin is well- known for its roles in both animals and humans, its presence and functional significance in plants were discovered later. The role of melatonin has also been identified in plants, related to stress—especially oxidative balance and in metabolic adaptation to adversity. Studies suggested plants that are treated with melatonin are capable of improving drought tolerance, in part through enhanced antioxidant action and enhanced control of stress-responsive pathways (Shreya et al., 2022; Karimian et al., 2025). Practically, these findings offer valuable insights for agricultural systems in areas where drought stress is a recurring constraint. Effective melatonin treatment may improve drought tolerance, thereby reducing yield losses during water scarcity. This is useful for retaining productivity under the most variable conditions in today’s climate. Melatonin could reduce reliance on synthetic chemicals that the industry uses frequently to treat crop stress, as it is a naturally occurring molecule. It is in line with today’s efforts to move more to environmentally conscious and sustainable farming. Though, melatonin application in agriculture is not without issue. Of note, plant responses are notoriously variable. Not all crops respond in the same way, and differences between cultivars have been reported, even within a single species (Maurya et al., 2025; Karimian et al., 2025). This variability makes it difficult to translate findings from controlled environments to field applications.

Understanding the best application technique, timing, and concentration is crucial to maximizing the agricultural effectiveness of exogenous melatonin in field settings, even though these factors are strongly influenced by crop stage and environmental factors. Foliar spraying, soil application, and seed treatments have all been effectively validated as application methods (Khan et al., 2020; Hasnain et al., 2023). Pre-sowing treatment, also known as seed priming, greatly increases seedling vigor indices and reduces initial drought-associated damage, making it a very successful timing strategy for building early resistance (Guo et al., 2022). Studies suggested that micronutrients foliar spray works best when applied during active vegetative or reproductive growth, especially for field-grown crops that are subjected to coupled abiotic challenges like heat and drought (Kumari et al., 2019, 2021). For the majority of commercial crop species, empirical evidence consistently indicates an ideal concentration range of 50 to 150 µM (or ~100 µmol·L^-^¹) across a variety of delivery techniques (Fu et al., 2023). The efficacy is enhanced at low doses of melatonin whereas high doses do not increase tolerance and may with times can interfere with normal growth. Finding suitable concentrations for different crops and stress levels is still a problem. In addition, the long-term environmental influence of chronic melatonin use should be evaluated with caution. Its persistence in soil or water and its likely influence on soil microbes or other organisms was not immediately clear (or still are unclear). There are also policy/regulatory considerations and economic considerations that must be taken in account. In some regions, melatonin may require regulatory approval for agricultural use. Large-scale application will also depend on its affordability for farmers, including production and application costs. At present, practical use may remain limited due to unclear guidelines and the high cost of preparation. Future studies should also integrate advanced molecular and omics-based approaches to unravel the mechanisms involved in biosynthesis, signaling pathways, and downstream regulatory networks. In particular, CRISPR/Cas-based gene editing offers a powerful strategy to validate the functional roles of key melatonin biosynthesis genes and signaling components. Additionally, transcriptomic and proteomic analyses can provide comprehensive insights into gene expression dynamics, protein regulation, and metabolic pathways involved in melatonin-mediated drought responses. The integration of these high-throughput technologies will facilitate the identification of critical regulatory networks and enable targeted genetic improvement of crops with enhanced drought resilience.

## Conclusions

Drought is a key barrier to agricultural production, leading to extensive crop losses and global food shortages. In this review, we investigated the crosstalk of melatonin with other phytohormones during drought stress response in plants, highlighting its modulation by and possible benefits of future study. Melatonin and other plant hormones are still not effectively regulated. Future approaches focused on phytohormonal contributions to the improvement of plants may be building plants for a stronger drought stress tolerance through direct targeting of melatonin interactions. Accordingly, this endeavor would provide comprehensive insight on the biochemical, morpho-physiological and molecular mechanisms of drought stress resistance in plants. Melatonin has an identifiable capability as a drought responsive plant enhancing treatment agent, while it is currently relatively underutilized in agriculture. More detail on its molecular mechanism, crop-specific action, and field performance is required. Melatonin should be applied for effective crop protection only after well-designed field trials, followed by rigorous scientific evaluation and proper agronomic management.
